# Sanitariums

**Published:** 1915-11

**Authors:** 


### Sanitariums.

Dr. Baty of McCaulley has purchased the old primitive Baptist church building, and will convert same into a sanitarium as soon as he can arrange the rooms, for treatment of all chronic diseases.

The joint city and county hospital of Wichita Falls is practically completed and will be opened to patients about October 15. The city council and county commissioners select the hospital board. The law provides that this board shall include two physicians, two laymen and one woman.

The Hotel Dieu of Beaumont has just been equipped throughout with new furniture by the B. Deutser Furniture Company, and is one of the most modernly equipped hospitals in the South.

A sanitarium for the treatment of cancer was opened at Mineral Wells, Texas, October 1st, with one of the most noted cancer specialists in the United States in charge. This institution bids fair to be of great importance to Mineral Wells, as the waters there will be of great service in the treatment of cancer, as well as nearly all constitutional diseases.

A new tubercular sanitarium is to be operated in the suburbs of El Paso as a result of a deal closed recently by Dr. James M. Laws, of Lincoln, N. M. The sanitarium in question will be modern throughout, and it is the purpose of Dr. Laws to operate a tent system whereby many of his patients will sleep in tents and enjoy the curative properties of fresh air.

A new and modern sanitarium is being installed at Floresville by Dr. J. F. Beckmeyer and his wife, Dr. Laura Beckmeyer, of San Antonio. Miss Gardiner, a graduate nurse from Kenney's Sanitarium, will hold the position of head nurse in this institution.

Dr. W. I. M. Smith of Nacogdoches has traded certain property to Dr. A. E. Sweatland for the Nacogdoches Surgical Hospital. Dr. Sweatland will continue to practice in connection with the hospital.

